# Reflection-mode TERS on Insulin Amyloid Fibrils with Top-Visual AFM Probes

**DOI:** 10.1007/s11468-012-9385-x

**Published:** 2012-06-15

**Authors:** Manola Moretti, Remo Proietti Zaccaria, Emiliano Descrovi, Gobind Das, Marco Leoncini, Carlo Liberale, Francesco De Angelis, Enzo Di Fabrizio

**Affiliations:** 1 Istituto Italiano di Tecnologia, Via Morego 30, 16163 Genova, Italy; 2 BIONEM Lab, University of Magna Graecia, Campus S. Venuta, Germaneto, viale Europa, 88100 Catanzaro, Italy

**Keywords:** TERS, Amyloid fibrils, Top-illumination setup, Field enhancement, Raman spectroscopy

## Abstract

Tip-enhanced Raman spectroscopy provides chemical information while raster scanning samples with topographical detail. The coupling of atomic force microscopy and Raman spectroscopy in top illumination optical setup is a powerful configuration to resolve nanometer structures while collecting reflection mode backscattered signal. Here, we theoretically calculate the field enhancement generated by TER spectroscopy with top illumination geometry and we apply the technique to the characterization of insulin amyloid fibrils. We experimentally confirm that this technique is able to enhance the Raman signal of the polypeptide chain by a factor of 10^5^, thus revealing details down to few molecules resolution.

## Introduction

Tip-enhanced Raman spectroscopy (TERS) is one of the most advanced tools for chemical characterization of materials down to nanometric resolution. This technique allows the confinement of electromagnetic radiation in very small volumes (few thousands of cubic nanometer), namely strong electric field enhancement. Nanofabricated metallic (or metal coated) tips are used to confine the electromagnetic radiation, while raster scanning the sample surface for a morphological, topographical, and optical characterization at the nanometer scale. Samples investigated by means of this method span from material science nanostructures to biomolecules and cells. Examples of material characterizations are semiconductors [1–3] and carbon derivatives [4–7]. Similarly, TERS is applied also for the investigation of organic and biological samples like organic molecules [8], collagen and amyloid fibrils [9, 10], nucleic acids [11], viruses[12], lipid bilayers [13], complex proteins [14], biofilms [15, 16], and cell membrane surfaces [17, 18]. Particularly, when dealing with either biosamples with small Raman cross-section or nanostructures with dimensions of few nanometers, the critical issues are the power of the enhanced field and its spatial localization around the tip apex. As a general “rule of thumb”, the sharper the tip the more confined is the field leading to several approaches for new kinds of TERS probes. Commonly, TERS tips are fabricated in order to exploit phenomena such as lightning rod effect, excitation of localized surface plasmon polaritons and resonant antenna excitation [19–24]. Furthermore, adiabatic compression was also recently exploited for TERS imaging [25–27]. In essence, a tip needs to be metallic or metal-coated in order to produce a local field enhancement effect upon illumination by a focused laser beam. Typical scanning tunneling microscopy probes are metal (Ag and Au) etched tips that can provide a controlled apex dimension with a quite reproducible technique [28, 29]. Differently, a TERS tip is based on atomic force microscopy (AFM) setups, which need a probe/cantilever system capable of working at suitable resonant frequencies. Silicon probes with a pyramidal tip coated with silver or gold are commonly used, together with metal-coated silicon oxide tips that have also been successfully employed for scanning near-field optical applications [25, 30–32]. Recently, due to the development of reflection mode TERS systems, silicon probes with a protruding tip have been developed (top visual probes). In our tips, the enhancement is based on the lightning rod effect and the excitation of plasmon polaritons at the tip apex is strongly dependent on local protrusions on the tip itself. In all cases, the nanofabrication procedure permits to calibrate the enhancement effect at will by intervening on the tip apex dimension. Depending on the experimental needs, constraints, and requirements, several TERS setups have been developed in the past. Different setups involve different light illumination geometries, such as: bottom, side, and top illumination type. Historically, the first used setup was based on the bottom illumination configuration and the collection of the scattered light from the sample was realized by an oil immersion objective lens [20, 21]. Ought to the high numerical aperture objective of the lens being used, the excitation spot was well confined and the collection less affected by background, even though limited by the use of transparent samples. Soon after, a side illumination objective was adopted allowing the analysis of opaque samples [22, 33]. However, the setup efficiency was limited by the low numerical aperture (0.28–0.55 NA) of the objective lens (small collection angle) and by the increased illumination area due to the angle between the focal plane and the sample surface. Recently, the top illumination approach has been implemented [34, 35]. This arrangement has shown to be suitable for the investigation of opaque samples. In addition, the use of a parabolic mirror for light illumination and collection further improves the efficiency of the system because of NA = 1 [36]. As a drawback, the alignment of the laser with the tip is difficult and prolonged, hence long working distance NA = 0.7 objectives lens are often preferred to focus the laser on the tip apex [34, 35].

In this work, we report on the application of a top illumination AFM setup for TERS measurements of nanostructured biological samples composed by insulin amyloid fibrils (IAF). Amyloids are the misfolded proteins existing in human organs, which lead to the formation of insoluble amyloid fibrils and plaques. The accumulation of fibrillar structures in tissues and organs may cause serious human diseases, generally known as amyloidosis, related to the neural disorder and other pathologies [37]. In particular, accumulation of insulin amyloid aggregates appears to be related to localized tumorigenesis in diabetic patients [38]. Characterization of the chemical structure of these aggregates is an important step towards the understanding of their formation process. High-performance bio-nano-analysis tools usually do not provide chemical data combined with topography resolution [39]. On the contrary, a technique like AFM/TERS allows to advantageously coupling chemical Raman data with the topography at a nanometric resolution. To date, there are few reports about TERS measurements on biological fibrils (collagen, β-amyloid, and insulin [9, 10, 39]). Deckert-Gaudig shows Raman data acquired in a transmission TERS setup on insulin fibrils deposited on glass with a gold-coated TERS tip. Here, we demonstrate that a top visual AFM probe in a top illumination setup is efficiently providing enhanced Raman spectra of IAF with a cross-section of less than 4 nm. We discuss the microRaman data compared with the TER spectra and find a range of wavenumber shifts due to a combination of the nanometric volume of the enhanced field at tip apex and the variety of molecules excited on the fibrils.

### Field-Enhancement on Nose-Type Gold-Coated Silicon-Based AFM Tips

**Fig. 1:**
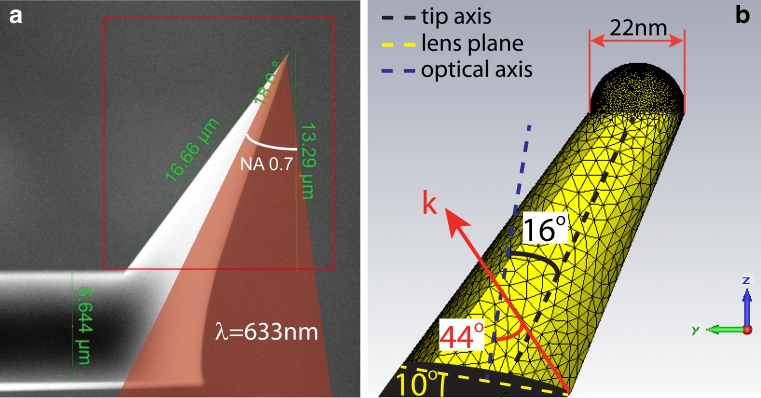
**a** SEM image of the nose-type gold-coated AFM tip. The *red* frame indicates the simulated part of the tip. **b** Mesh representation of the tip end. The spherical ending (11 nm radius) was taken off axis in order to better represent the roughness of a gold-coated tip. The *red shadow* indicates laser focus (out of scale) The 44° half-cone light is associated to the 0.7 NA lens used in the experiment (see “Materials and Methods” section)

We have performed a fully 3D calculation of a nose-type gold-coated AFM tip. In particular, the chosen structure is 16 μm long with a tip radius of curvature of 11 nm. These geometrical parameters were obtained from the scanning electron microscope (SEM) images of the TERS tips used in the actual experiments. The gold optical properties [40] were implemented according to a full Drude–Lorentz description. The source was set at *λ* = 633 nm with an incident angle about 16° off the axis of the structure, corresponding to our experimental condition where an incident source tilted 10° from the normal to the surface of the cantilever was implemented (see “Materials and Methods” section). The chosen linear polarization was mainly transversal to the tip axis to match the experimental conditions (in our coordinate system, almost parallel to the *y*-axis). The SEM image of the fabricated tip and the tip-end of the simulated structure are shown in Fig. 1a and b, respectively.

**Fig. 2:**
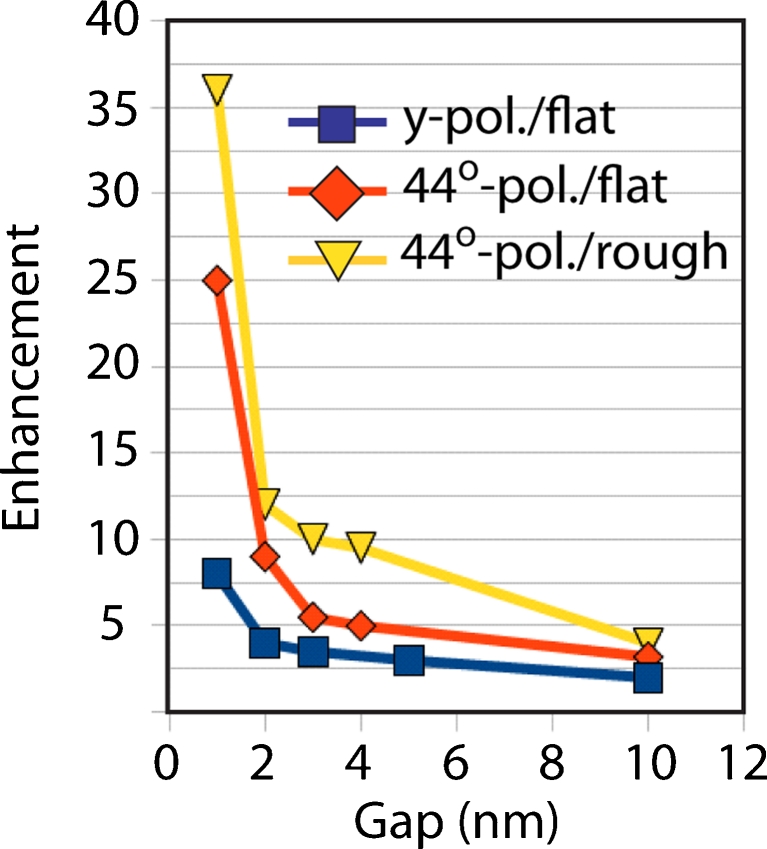
Electric field enhancement versus tip–substrate gap. The radius of curvature of the tip end is 11 nm, with a source of wavelength 633 nm polarized mainly along *y*-direction. The three curves are associated to two different configurations: *curves red*/*blue square* represents the field enhancement of a tip flat substrate system for two different polarizations. The *yellow triangle curve* is instead associated to a configuration where the substrate shows local roughness

**Fig. 3:**
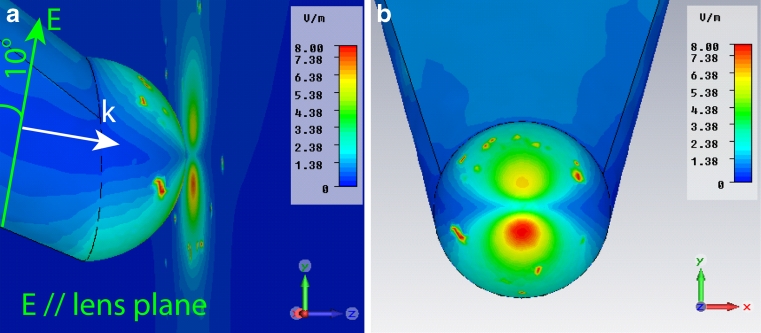
Electric field enhancement for a tip–substrate gap equal to 1 nm. The radius of the tip end is 11 nm. The substrate is considered to be flat in order to avoid any SERS contribution to the field enhancement. A strong *y*-oriented dipole behavior is observed, which is consistent with the polarization direction. The localized red spots are mesh singularities. The two figures represent the total (tip and substrate) view and the tip only view, respectively. *K* vector and electric field of incident radiation are indicated

A set of simulations was performed in order to determine the electric field enhancement provided by the tip when applied on insulin amyloid fibrils. To take into account the presence of the fibrils, we have modified the refractive index of the volume around the tip to 1.4. In particular, a gold-coated flat substrate was approached to the tip within a gap ranging from 1 to 10 nm. The choice of an ideally flat substrate was to remove any surface-enhanced Raman scattering (SERS) contribution to the field enhancement originating from the substrate itself. A second geometrical configuration was also implemented. In this case, the TERS tip was approached to a smooth spherical surface which simulates the local roughness of the surface. Also in this case, it was possible to avoid any SERS contribution to the field enhancement. Figure 2 plots the field enhancement versus the tip–substrate gap for the two configurations. It shows that only for distances far below 10 nm, a strong field enhancement is obtained. In fact, considering the polarization condition, the tip-end will behave like an isolated spherical object by creating a dipole-like field profile along the polarization direction [41]. The effect of the substrate starts emerging only for gaps below 2 nm in case of a flat substrate (blue/red square lines), and 4 nm if the local roughness is considered (yellow triangle line). In fact, below these values of the gap, the field enhancement is at least equal to 10, meaning a Raman signal of the order of 10^4^, which well approach our experimental results. Figure 3 is the 3D electric field enhancement distribution in the area around the flat substrate. The gap is 1 nm. It is interesting to observe the dipole-like shape of the electric field along the *y*-direction (*y*-pol/flat). The field enhancement is, at its maxima, around 8. This value is lower than the estimated experimental value, which is expected to be around 20 (see “Results and Discussion” section).

**Fig. 4:**
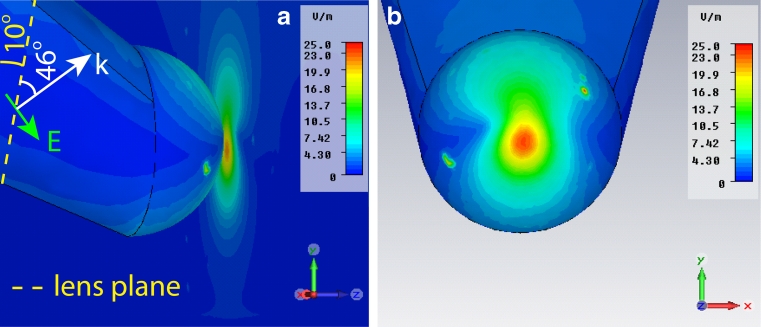
Electric field enhancement for a tip–substrate gap equal to 1 nm and 44° polarization. The field shows a single hot spot in the center of the tip. In fact, in the present case, the polarization is along the tip axis. An electric field enhancement of 25 is found. *K* vector and electric field of incident radiation are indicated

In order to explain this discrepancy, we have modified the polarization direction 44° off the previous case (from now on, we shall refer to it as the 44° polarization). In fact, in the real experiment, to focus the light on the structure a 0.7 NA lens was utilized. Hence, this polarization change is an approximation to take into account the effect of the lens on the beam. Figure 4 shows the electric field enhancement for a tip–substrate gap of 1 nm with 44° polarization. The enhancement is about 25 (see Fig. 2, 44° pol/flat), more than three times higher than in Fig. 3. The reason comes from the polarization direction, which is roughly parallel to the tip axis. In fact, the tip will behave like a giant dipole with dimensions much larger than the 11 nm radius spherical tip, generating a stronger electric field. In this way, a stronger field can be generated. Interestingly, the value of enhancement 25 is very similar to the experimentally estimated value even though the gap value of 1 nm is lower than the estimated fibril thickness, which, as we will see, is around 3 nm.

**Fig. 5:**
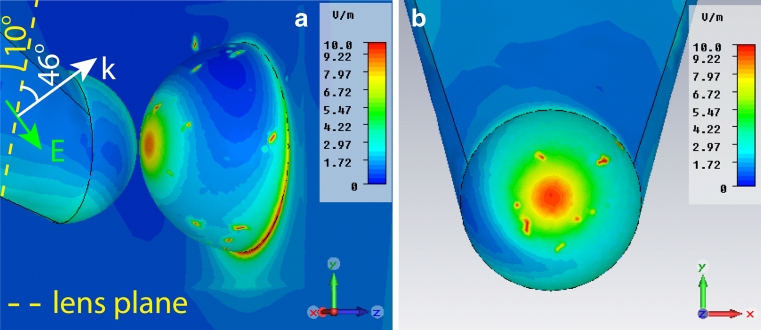
Electric field enhancement when a rough substrate is considered. The island aggregate has a 30 nm diameter. The gap is 3 nm, which returns a field enhancement around 10. *K* vector and electric field of incident radiation are indicated

Furthermore, as shown in Fig. 2, when the gap between the tip end and the substrate is increased over 1 nm, the simulations show a decay trend, similar to the case of polarization along *y*-direction. However, as expected, the 44° polarization shows always better enhancement than the polarization along *y*-direction. To be noticed that the electric field enhancement for a gap of 3 nm (fibril thickness) is just slightly above 5, which is lower than the experimental results (enhancement ∼20). We have then modified the surface of the substrate to take into account the local roughness. In fact, SEM images of the gold substrate (not shown) suggest a surface with local roughness associated to island aggregated with an average diameter of 30 nm. We have then implemented this feature in the simulations, as shown in Fig. 5. The results show a sensitive increase of the electric field enhancement to 10, which gives a Raman signal around 10^4^. Hence, the comparison between the two geometrical configurations suggests that the existence of local protrusions emerging from the substrate might well explain the Raman signal obtained in our experiments.

## Materials and Methods

In this section, details about the TERS probes employed and the experimental setup will be provided. Particular attention will be given to the problem of laser alignment with the tip.

### TERS Setup

**Fig. 6:**
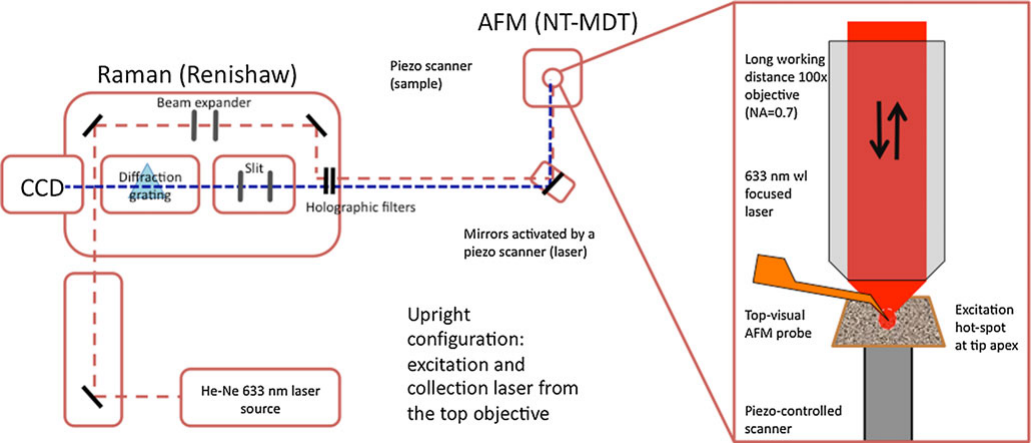
Experimental setup equipped with top illumination mode and zoom-in in the field enhancement area

A custom setup provided by NT-MDT (Zelenograd, Russia) was used. The setup is a combination of NT-MDT AFM and Renishaw MicroRaman spectrometer (Fig. 6). The apparatus is conceived for laser illumination of the probe from the top and for signal collection both in reflection mode and in transmission mode. Here, we used exclusively the backscattered signal collection mode (upright configuration). A He–Ne source provides a linearly polarized 632.8 nm laser light (12 mW) illuminating a long working distance objective (Mitutoyo Japan 100×, NA = 0.7) mounted within a proper AFM head. Light is focused within a spot whose diameter is about 600 nm. An adjustable cantilever holder hosting the TERS probe is placed close to the focal plane of the objective. Scattered and/or reflected light is collected by the same objective and sent to the dispersive spectrometer (grating 1,800 lines/mm) after being spectrally filtered through an edge filter (633 nm plus Rayleigh scattering).

AFM control is performed by means of a standard beam deflection method. A laser beam probing the cantilever bending is focused onto the cantilever by means of the top objective used for probe illumination. The AFM control beam is an 830 nm wavelength diode laser light. Topographic and spectral measurements are performed by raster scanning the sample beneath the illuminated tip. The probe is mounted on the holder with a 10° tilt angle between cantilever and sample surface. In order to align the Raman laser with the tip apex, the instrument was equipped with mirrors moved by a closed-loop piezo-controlled scanner that allows an accurate 3D positioning of the laser spot onto the tip.

### Laser Alignment

**Fig. 7:**
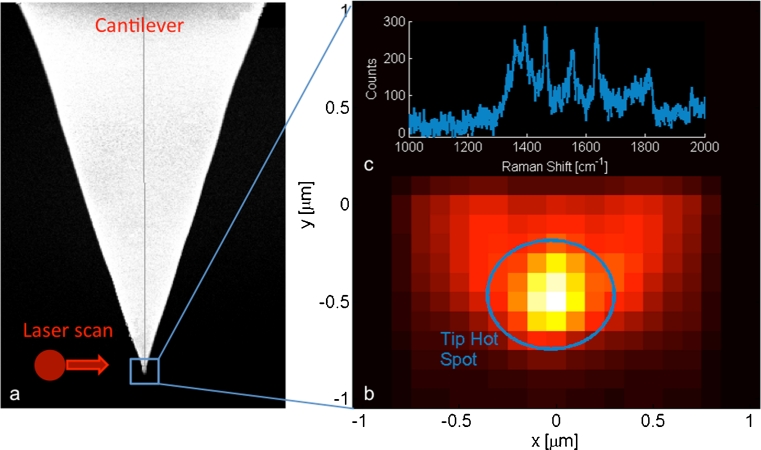
**a** SEM image of the nose-type probe tip and a sketch of the scanning laser beam; **b** intensity map of the backscattered laser as a function of the scanning beam position; **c** enhanced Raman spectrum of Rhodamine-6G corresponding to the “hot spot” in **b**

The laser alignment with the tip apex is a very critical issue when performing TERS measurements. In fact, quite often it is faced by trial and error approach, resulting in a time-consuming procedure. By adding a piezo-driven set of mirrors to a top illumination setup [42], the instrument is able to perform an *x*–*y* (being *z* the vertical direction) laser scan on the tip itself to facilitate the location of the so-called “hot spot”. The hot spot is generated at the tip apex by an enhancement of the electromagnetic field. The laser scan can be performed both to find Rayleigh scattering of the laser and to find Raman scattering enhancement from the tip. However, the latter is more accurate in order to perform the best match between tip and laser for TERS measurements. To exemplify this procedure, we used a gold substrate covered with Rhodamine-6G (Sigma) to find the best alignment between laser and tip apex. While the cantilever is held at a suitable amplitude oscillation on the substrate, an *x*–*y* laser scan (Fig. 7a) of 32 points by 2 μm (at 5 % laser power for 1 s/point) upon the protruding tip was performed. For each position of the illuminating spot, the whole Raman emission and the backscattered laser light are collected and spectrally analyzed. In Fig. 7b, a false color image of the backscattered laser signal is presented as a function of the scanning beam position. Within the scanned area, we were able to identify an optimum beam position leading to a corresponding intensity maximum of the backscattered laser (blue circle in Fig. 7b). Associated to this “hot spot”, a Rhodamine-6G enhanced Raman spectrum is found [43], as illustrated in Fig. 7c.

### TERS Probes

Commercial silicon AFM probes (Vit-P top-visual probes, NT-MDT) for tapping mode AFM (resonance frequency around 300 kHz) were thermally evaporated with a thin layer of gold (25 nm) at a 30° angle to the source and used as such for TERS experiment. Probes were stocked under vacuum and used within few days after the gold coating.

### Sample Preparation

Gold-coated silicon wafer was used as the substrate for all TERS experiments. A 50-nm gold layer was sputtered on the substrate. Deposition of IAF on gold-coated substrate was made without additional preparation. Mature amyloid fibrils were obtained from bovine insulin (5 mg/ml; Sigma-Aldrich). Protein solution was incubated in HCl water solution (MilliQ filtered water) at pH 2.00 for 3 weeks at 60 °C. A drop of the preparation as such was pipetted directly on silicon substrate for acquisition AFM image. For TERS measurements, a drop of 20 μl of a 1:10 diluted solution was deposited onto the substrate. After 1 min, the sample was rinsed with 5 ml of MilliQ water, air-dried, and put on the AFM sample holder as such.

## Results and Discussion

In this section, we present the AFM topography and point TERS measurements of a monolayer of IAF deposited on gold coated silicon substrates. The first step is to acquire a topography image to locate the IAF that are monodisperse on the substrate. For TERS measurements, the oscillation amplitude of the tip is set at less than 10 nm [44]. Once the suitable fibril is found, the tip is kept at a fixed position and a laser scanning collecting full-spectrum Raman data is performed to find the enhancement spot at the tip apex (see laser alignment in “Materials and Methods” section). Upon finding notable points, the corresponding TERS spectra are collected at 1 s time/point and 1 % laser power moving the sample stage to the desired position. Offset for Raman shift spectra is set at the 520.1 cm^−1^ corresponding to the peak of the first order Si–Si phonon band [45]. Additionally, a reference microRaman spectrum on sputtered gold substrate of bulk IAF was acquired for peak comparison.

**Fig. 8:**
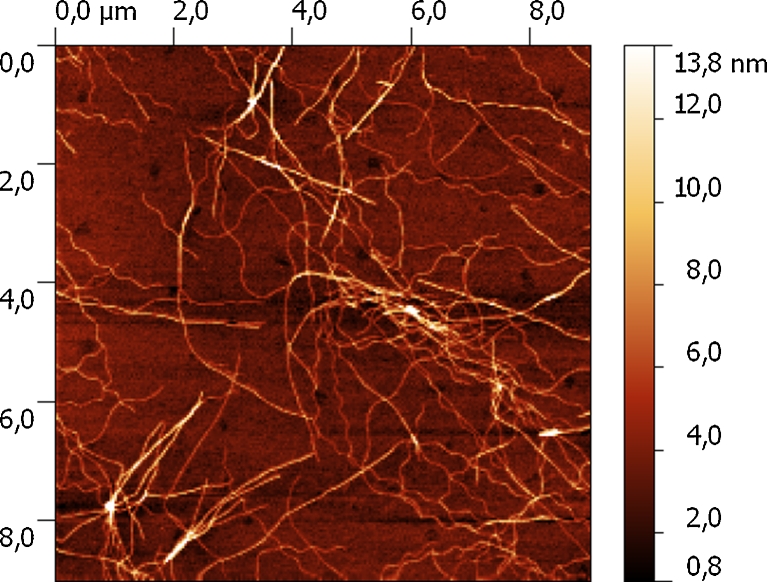
AFM topography of insulin amyloid fibrils deposited on silicon substrate

The image shown in Fig. 8 is an example of AFM topography acquired on an area covered with dispersed IAF deposited on a silicon substrate. IAF fibrils can be arranged in different forms, such as ribbon- or twisted-like wires, which can exhibit further orders of aggregation of increasing complexity. Topographically, this results in a number of IAF populations showing a variety of heights. After a careful analysis of AFM data, we could estimate that the simplest IAF aggregates detected show a mean height value of roughly 3 ± 1 nanometers.

**Fig. 9:**
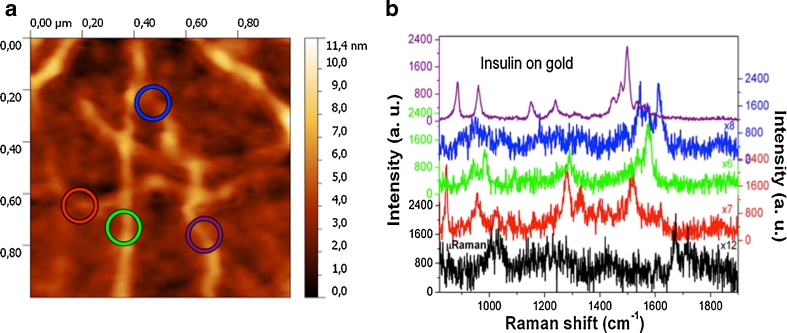
**a** AFM topography of insulin fibrils (IAF) on gold-coated silicon substrate and related Raman spectra at color-coded related positions. **b** MicroRaman spectrum (*black*), TERS spectra (*red*, *green*, *blue*, and *purple graphs*). For better comparison, spectra are multiplied by the indicated factors

Combined AFM-TERS measurements were performed on gold-coated silicon substrates, covered with a monolayer of IAF. Figure 9a shows the surface morphology of the IAF/gold structure. The four circles represent the spatial spots providing the TERS (near field) signals, which were compared with microRaman (far field) measurements in order to estimate the working properties of the TERS tip. Various characteristic Raman bands, distributed in the range of 800–1,800 cm^−1^, were observed, each associated to different amino acids and to the conformational structure of the amyloid protein. A number of vibrational bands, intensity, and width for TERS and microRaman spectra are reported in Fig. 9b. In particular, the far-field spectrum of IAF (black line) shows various peaks, centered at 1,003 cm^−1^ (phenylalanine), 1,035 cm^−1^ (phenylalanine), 1,675 cm^−1^ (secondary structures). In the blue TERS profile, a small contribution of a peak at around 1,690 cm^−1^, related to the secondary structures is visible. Furthermore, the Raman bands related to single amino acid features such as at 1,610 cm^−1^ (tyrosine aromatic side chain), 1,574 cm^−1^ (phenylalanine), 1,546 cm^−1^ (tryptophan), 1,499 cm^−1^ (CHx asymmetric bending), 961 cm^−1^ (ring stretching), 885 cm^−1^ (tyrosin side chain), and 846 cm^−1^ (tyrosin) were found to be very much enhanced in the TERS spectra [46, 47]. Particularly, the spectrum corresponding to the purple hot spot shows a very neat and clear profile. Hence, the combination of the TERS spectra provides both higher intensity and number of bands than the far-field microRaman analysis, namely better chemical description of IAF samples.

To be noticed, the intense peaks in the near-field measurements are observed predominantly for aromatic groups such as tryptophan, tyrosine, and phenylalanine components due to both the strong electric field enhancement and the favorable orientation of the aromatic amino acids (in accordance with [10]). By observing the four TERS spectra in Fig. 9b, it can be noticed that both the spectral intensities and the spectra profiles are strongly related to the corresponding spatial locations. These differences originate from the alteration of protein orientation at those particular positions. These findings made clear observations, which also confirm the detection of protein in the limit of few molecules. The far-field Raman signal, collected from the same measurement area but with the tip retracted, did not show any appreciable peak, with counts comparable to background noise of the instrument. We therefore cannot precisely estimate the field enhancement by comparing near- and far-field measurements as reported in [19], but we can retrieve the minimum field enhancement required to obtain our TERS signal levels. This can be done by supposing the far-field Raman signal level to be equal to the instrument noise level, and then applying the formula [19]:

$$ \frac{{S_{\text{TERS}}}}{{S_{\text{FF}}}} = \frac{{A_{\text{TERS}}}}{{A_{\text{FF}}}}{g^4} $$

where *S*_TERS_ is the TERS signal level, *S*_FF_ is the far-field Raman signal level (tip retracted), *A*_TERS_ and *A*_FF_ are the illuminated areas in TERS and far-field configuration, respectively. Then, by considering *A*_TERS_ ≅ 120 nm^2^, *A*_FF_ ≅ 10^4^ nm^2^, we obtain a minimum field enhancement factor (*g*) in the order of 20.

## Conclusion

A plethora of configurations for TERS measurements is emerging due to encouraging results obtained by combining high-resolution AFM topography with Raman spectroscopy characterization. AFM analysis is nowadays a solid and well-developed technique that guarantees a subnanometric topography resolution. Moreover, the continuous improvement in the fabrication of suitable probes allows easy access to TERS tips. In this research work, we used a top illumination and collection TERS approach to study a biological sample of medical interest by a top visual gold-coated probe. Amyloid fibrils are particularly suitable for TERS measurements because they conjugate a small Raman cross-section with a well-defined nanostructure, already well classified by standard AFM measurements. Thus, after finding the fibril with the suitable height for analysis, we were able to obtain a Raman enhanced emission on selected spots along the fibril by exploiting the tip enhancement effect on rough gold-coated substrates on which insulin fibrils are deposited. Simulations performed with a quasirealistic configuration, confirm the occurring of a strong field enhancement (10) effect corresponding to our configuration. Ought to the large field enhancement focused in a nanometric area, TERS collected spectra showed Raman signal enhancement (∼10^5^) with remarkable peaks both displaying the supramolecular organization of the fibrils and, noticeably, the orientation of few molecules in different points along the fibrils.
